# Burden and distribution of chronic kidney disease in sub-saharan africa: a systematic review with meta-analysis

**DOI:** 10.4314/ahs.v25i1.29

**Published:** 2025-03

**Authors:** Martins Nweke, Theresa Ado-Aghughu, Tobi Daniels, Uzunma Imo

**Affiliations:** 1 Evangel University Akaeze, Physiotherapy; 2 David Umahi Federal University of Health Sciences, Physiotherapy; 3 University of Pretoria, Physiotherapy; 4 University of Benin, Physiotherapy

**Keywords:** Chronic kidney disease, sub-saharan africa, systematic review, meta-analysis

## Abstract

**Background:**

Chronic kidney disease (CKD) is fast becoming a leading non-communicable disease in sub-Saharan Africa. Efforts directed at mitigating CKD will thrive on precise and accurate estimation of CKD burden, which often varies widely owing to study characteristics like methods of estimating Glomerular Filtration Rate (GFR) and study population.

**Objective:**

To determine the burden of CKD and distribution of this burden in sub-Saharan Africa based on study characteristics.

**Methods:**

Involved systematic review of articles peer-reviewed literature published in English. Review was conducted consistent with Preferred Reporting Items for Systematic Reviews and Meta-Analyses checklist. Data sources for review include MEDLINE, PubMed, CINAHL, Academic Search Complete, African wide information and articles that reported prevalence of chronic kidney disease in sub-Saharan Africa. Bias risk assessment was conducted using mixed-method appraisal tool. Random-effect model of meta-analysis was employed to quantify effects of variation study characteristics on burden of chronic kidney disease in sub-Saharan Africa.

**Result:**

Showed statistically significant difference in CKD prevalence by study population (F=2.547, p=.019) and epidemiological significance difference in GFR estimate method with Schwartz approach (35%).

**Conclusion:**

CKD remains a public health issue in sub-Saharan Africa and the distribution varies widely according to region, study population and method of estimating GFR.

## Introduction

Chronic kidney disease (CKD) is a major health problem in sub-Saharan Africa, with a variety of risk factors including communicable and non-communicable diseases^1^. Rapid urbanization, HIV epidemic, and rising non-communicable disease rates are all contributing to the susceptibility of chronic kidney disease in Sub-Saharan Africa^1^. It's part of a new chronic disease epidemic that has overtaken starvation and infection as the leading causes of mortality in the twentieth century^2^. CKD more than doubled as a cause of death between 1990 and 2010, rising to the 18^th^ most common cause of death worldwide^3^ with a surge in prevalence second only to HIV and AIDS^4^. By 2030, more than 70% of patients with end-stage renal disease are expected to reside in low-income nations where poverty is prevalent^5^,^6^. Several studies have been undertaken in Sub-Saharan Africa to estimate the prevalence of CKD.

Stanifer et al.^7^ published a systematic review in which they estimated its prevalence of 13.9%. Previous studies that documented CKD prevalence in the general population/comorbid illnesses known to induce renal impairment like hypertension, diabetes, and HIV were included. Despite heterogeneity in study characteristics, notably the method for determining Glomerular Filtration Rate (GFR), the authors performed a meta-analysis to generate a 13.9% estimate without accounting for this potential confounder. In sub-Saharan Africa, differences in study characteristics like GFR measuring techniques and study population contribute significantly to bias in assessing the burden of CKD1. For example, the prevalence of CKD was optimized to 24.4%-26% using the Cockcroft–Gault equation, whereas MDRD and CKD-EPI equations yielded prevalence estimates of 2.5–12.3 per cent and 11.4 per cent, respectively^1^. The implementation of cost-effective early detection programs and reliable assessment of the severity of the problem is crucial for the management of CKD epidemics in Africa^1^. Failure to analyze the impact of methodological variations on CKD burden and distribution will invariably result in erroneous estimates and, consequently, little confidence in synthesized estimates, thus hampering policy-making and implementation. Therefore, the goal of this study is to evaluate the burden and distribution of CKD in sub-Saharan Africa. Specifically, the study determined the burden of chronic kidney disease and its distributions based on selected study characteristics in sub-Saharan Africa.

## Methods

This is a systematic review of observational studies, comprising retrospective surveys, cross-sectional studies, and cohort studies. The protocol was structured using a hybrid of the PRISMA checklist and Meta-analysis of Observational Studies in Epidemiology (MOOSE) guideline. The protocol was registered with PROSPERO (ID: CRD42022299774). This review included observational studies of epidemiological design written in English.

Where appropriate, articles written in French were translated to English using Google translator. Eligible studies were selected irrespective of sample size and test statistics. Studies were conducted within sub-Saharan Africa. This review included only studies in which participants' CKD was diagnosed via GFR estimation, using standard procedure. Operationally we defined chronic kidney disease as the presence of kidney damage or an estimated glomerular filtration rate(eGFR) less than 60ml/min per 1.73 square meters. We included studies irrespective of whether the study had a control group or not and studies irrespective of whether the population is of high risk or not, but this was properly accounted for during data synthesis. For each study, we assessed the prevalence of CKD, method of GFR estimation, disease status (e.g. hypertension, diabetes, HIV status), age, sampling method, smoking, sub-Saharan region, assessors' qualification, and alcohol and substance use. Inclusion criteria were medium to high-quality studies that reported the prevalence of CKD and stated the method of GFR measurement. Exclusion criteria included systematic review and/or meta-analysis, low-quality studies, unclear GFR measurement methods and CKD prevalence reports conducted outside sub-Saharan Africa.

We searched the literature using several combinations of search terms from medical subject headings (MeSH) and keywords in the title, abstract, and/or text of articles. Search covered the inception of the databases to December, 2021. First, we did a pilot search in PubMed to establish the face sensitivity of the search strategy (table 1). The PubMed pilot search included various MeSH terms generated using Cochrane Mesh finder. Terms were adapted to syntax and subject headings of remaining databases. Finally, we searched the remaining databases: PubMed, MEDLINE, Academic Search Complete, CINAHL, African-wide Information and reference lists of identified observational and review articles for relevant studies. EndNote 20 was used to de-duplicate all literature search results. The remaining articles were exported to EndNote 20 for further de-duplication, and independent screening of titles, abstracts, and full-texts. Screening forms with questions regarding eligibility were developed, piloted, and refined to make the screening process more efficient.

**Table 1:** PubMed Pilot Search Strategy

Search terms	Database	Date of search	Filter	No. retrieved
Kidney Insufficiencies, Chronic OR Kidney Insufficiency, Chronic OR Chronic Renal Insufficiencies OR Chronic Kidney Insufficiency OR Renal Insufficiencies, Chronic OR Chronic Renal Insufficiency OR Chronic Kidney Insufficiencies OR Renal Diseases, Chronic OR Kidney Diseases, Chronic OR Chronic Kidney Disease OR Disease, Chronic Renal OR Chronic Kidney Diseases OR Chronic Renal Diseases OR Renal Disease, Chronic OR Diseases, Chronic Renal OR Chronic Renal Disease OR Disease, Chronic Kidney OR Diseases, Chronic Kidney OR Kidney Disease, Chronic[MeSH Terms]) AND (prevalence OR burden of illness OR epidemiology[MeSH Terms])) AND (Filtration Rates, Glomerular OR Filtration Rate, Glomerular OR Rate, Glomerular Filtration OR Glomerular Filtration Rates OR Rates, Glomerular Filtration[MeSH Terms])) AND (sub-Saharan Africa OR Africa, sub-Sahara[MeSH Terms])	PubMed	18/12/2021	None	176

The outcome of the full-text review was subjected to data extraction by the student reviewer and verified by supervisor reviewer. The outcome of screening was reported using a PRISMA flow diagram.

### Data Collection Process

#### Quality appraisal and risk of bias assessment

We used the Quality assessment checklist for prevalence studies, adapted from Hoy and colleagues^10^ to assess quality and each article bias risk. Appropriateness and adequacy of methodology, study design, participant recruitment, data collection, data analysis, and presentation of findings were examined by the checklist. It is suitable for appraising most studies reporting prevalence. The tool comprises nine questions and a summary score. When there was insufficient evidence to assess the risk of bias, studies were labeled as unclear. A third reviewer compared, completed, and collated results after two research assistants independently assessed the risk of bias.

#### Data items

Author affiliations, participant characteristics, inclusion criteria, exclusion criteria, study sample size, sampling methods, high/low-risk population, diagnostic criteria/method of assessment, the prevalence of CKD, method of GFR estimation, hypertension, diabetes, HIV status, age, country, region, and assessors qualification/experience were all collected from each article.

#### Data synthesis and assessment of heterogeneity

To determine pooled prevalence estimates of CKD estimates for different measuring instruments and categories of assessors, we utilized a random-effects meta-analysis model^11^,^12^. Narrative display of measures of heterogeneity, i.e. study characteristics was sorted by year of publication and presented in an evidence table (Table 2). The measures of heterogeneity, namely Cochrane's Q statistics, and I2 were computed in line with Higgins and colleagues^13^.

**Table 2:** Socio-demographic and Study Characteristics

Variable	Mean	Standard deviation
Age	34.2	20.2
% female	58.7	16.2
Sample size	818.2	1254.3
**Design**		
Cross-sectional	96	80
Cohort	24	20
**Sampling technique**		
Random	37	30.6
Non-random	84	69.4
**Study population**		
HIV	24	20.0
Apparently healthy	52	43.3
Mixed patients	11	9.2
Diabetes	11	9.2
Sickle cell disease	5	4.2
Hypertension	13	10.8
Stroke	2	1.7
Diabetes &hypertension	2	1.7
Cancer	1	0.8
**Method of estimating GFR**	43	36.4
MDRD	36	30.5
CKD-EPI	29	24.6
Cockcroft-gault	7	5.9
Schwartz	3	2.5
MDRD&CKD-EPI	3	2.5
Others		
**Region**		
West Africa	59	48.4
East Africa	49	41.0
Southern Africa	9	7.4
sub-Saharan states	3	2.5
mixed	1	0.8

## Ethical consideration

Not Applicable.

### Data analysis

Comprehensive Meta-analysis version 3 and SPSS version 22 were used to conduct data synthesis and determine an estimate of pooled CKD prevalence and the effects of study characteristics on CKD prevalence estimates. We employed random-effect model of meta-analysis. Appropriately, independent-test/ANOVA evaluated the effect of each study characteristic on prevalence. The level of significance was set at 0.05. Univariate analysis was used to examine the effects of study characteristics on the prevalence of CKD while adjusting for all significant covariates in other to determine CKD burden. An increase or decrease in the prevalence of 7% was defined as epidemiologically significant to distinguish statistical significance from epidemiological significance. A covariate was considered significant if it accounts for ≥7% of the variation in prevalence^15^. The burden of CKD was calculated by multiplying the number of adults in sub-Saharan Africa by the estimated prevalence of CKD (with 95%CIs)^16^. Currently, 624, 523, 061 individuals aged 15 years and above reside in Sub-Saharan Africa^17^.

### Publication bias/Metabias

To examine meta-bias, we created a funnel plot and run Egger regression test^18^. Only studies available as abstracts were included in the analysis to determine if they affected the direction of effect size.

## Results

### Review profiles

**Figure 1:**
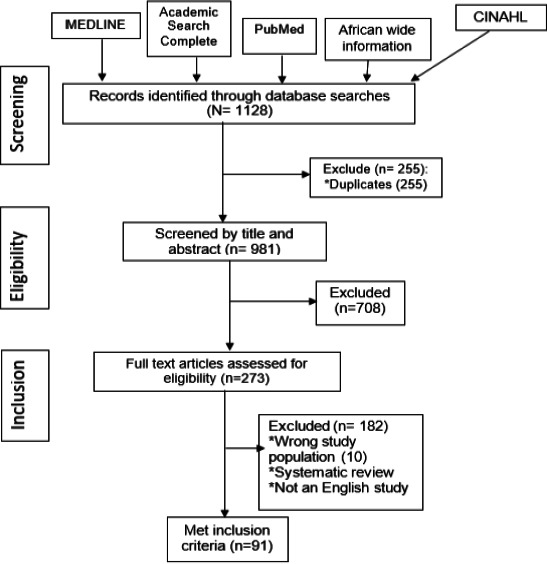
PRISMA Flow Diagram of the systematic review on the Effect of Variation in Methods of Glomerular Filtration Rate Measurement in Estimation of the Burden of Chronic Kidney Disease in Sub-Saharan Africa 2012-2021

We identified 1128 records. After duplicates were removed, 981 records remained. After title and abstract screening, we excluded 708 irrelevant records, leaving 273 articles for full-text screening. Ultimately, we included 91 articles involving 76,721 participants from 17 countries (Figure 1). Of the 91 included studies, we extracted 121 prevalence points because some studies presented more than one method of estimating GFR and hence more than one prevalence point. The 121 prevalence points were involved in the meta-analysis.

### Sociodemographic and Study Characteristics

The mean age was 34±20 years. Approximately 59% of the participants were females. Included studies were conducted amongst; 52 (43.3%) healthy individuals, 24 (20%) people living with HIV, hypertensive individuals (10.8%), 11(9.2%) diabetics, 5 (4.2%) sickle cell and 2 (1.7%) stroke patients. More than half of the studies (69.4%) employed non-random sampling and most (80%) of the studies utilized a cross-sectional design. Regarding methods of estimating GRF, MRDR was utilized in 43 (36%) studies, CKD-EPI was employed in 36 (31%) while 29 (24.6%) studies used the Cockcroft-Gault approach (Table 2). Of the 121 prevalence points, 48.4% and 41% were reported in West Africa and East Africa respectively (Table 2 and supplementary file 1).

### Prevalence and burden of CKD in SSA

**Figure 2:**
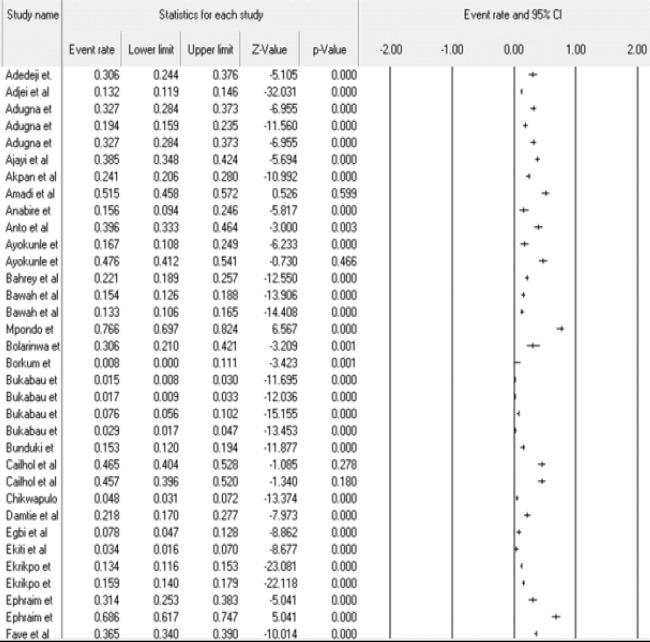

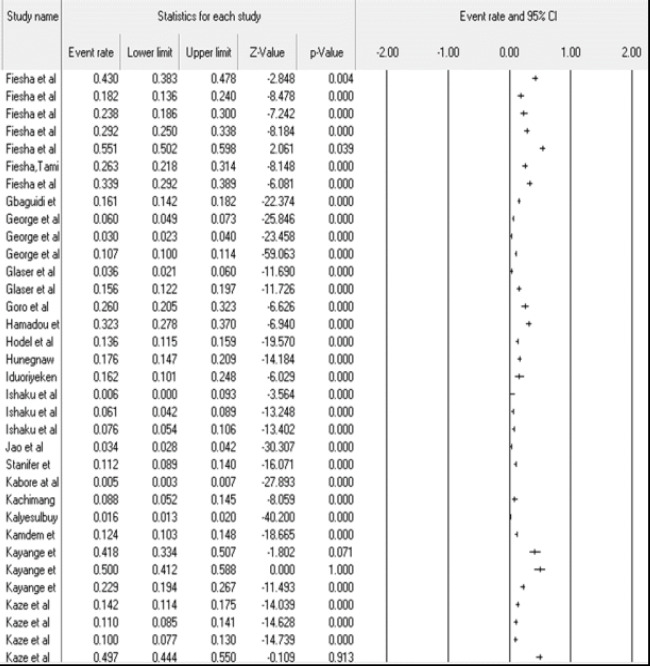

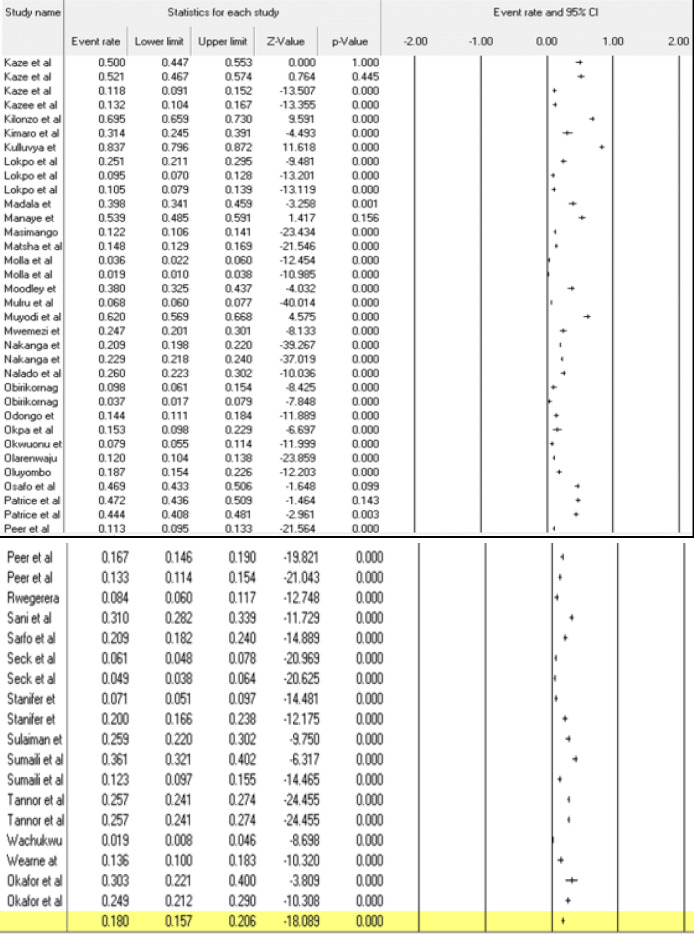
Forest plot displaying the prevalence of CKD in sub-Saharan Africa

**Figure 3:**
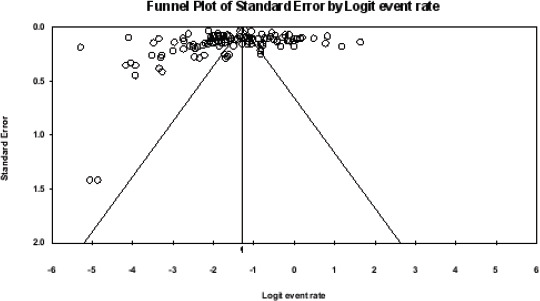
Funnel plot displaying publication bias

Result from the meta-analysis showed that pooled prevalence of CKD in sub-Saharan Africa was 18.0% (95% CI = 0.157 − 0.206), with a burden of 174, 031, 500. There was a substantial degree of heterogeneity (I2 = 98.74) (Figure 2). There was no publication bias (Egger's t-value= 1.2111, p = 0.2283) (Figure 3).

### Distribution by sociodemographic (age and gender)

Analysis of the influence of sociodemographic and study characteristics showed that there were statistically significant weak positive correlations between prevalence rate and age (r = 0.221, p = 0.015). There was no correlation between the CKD prevalence rate and between percentage number of females (r = -.058, p = 0.127) (Table 3).

**Table 3:** Effects of study characteristics on estimating the prevalence of CKD in sub-Saharan Africa

Study characteristics	Publication (N)	Prevalence %(CI)	r/t/F-value	p	Adjusted prevalence %(CI)	F	P
Age	121		.221*	.015			
Gender	119		-.058	.532			
Sample size	121		-.213*	.019			
**Study design**							
Cross-sectional	96	24.0 (20.4-27.6)	2.361	.127	25.1(21.7-28.5)	6.239	0.14
Cohort	24	17.8 (10.6-24.9)			14.7(7.3-22.1)		
**Sampling technique**			9.476	0.003		8.810	.004
Non-random	84	25.9 (21.8-30.1)			10.6 (21.7-28.5)		
Random	37	15.5(11.7-19.4)			-10.6(-17.7-3.5)		
**Region**							
West Africa	59	21.8(17.7-26.0)	1.385	.255	20.4 (15.9-24.9)	2.096	.128
East Africa	50	25.9 (20.1-31.7)			27.0(22.1-31.8)		
Southern Africa	9	16.6(6.1-27.0)			19.3 (7.4-31.3)		
**Population**							
HIV	24	25.0(16.1-34.0)	4.725	.000	27.6(20.4-34.8)	2.547	.019
Apparently	52	14.0(11.1-17.0)			17.0(12.1-21.9)		
healthy							
Mixed patients	11	35.1(23.3-46.9)			31.6(20.7-42.6)		
Diabetes	11	32.4(17.6-47.3)			32.6(22.4-42.8)		
SCD	5	40.3(20.1-60.4)			38.1(22.3-53.8)		
Hypertension	13	26.3(15.5-37.1)			21.1(11.6-30.7)		
Stroke	2	23.4(-8.0-54.8)			17.3(-5.5-40.2)		
Diabetes & hypertension	2	25.8(24.2-27.4)			19.8(-3.3-42.8)		
**Method of estimating GFR**						1.089	.366
MDRD	43	21.8(17.2-26.3)	.783	.539	21.0(15.7-26.2)		
CKD-EPI	36	20.6(14.4-26.8)			22.7(16.8-28.6)		
Cockcroftgault	29	25.9(17.6-34.3)			24.6(18.2-31.0)		
Schwartz	7	31.2(16.7-45.8)			35.0(21.1-49.0)		
MDRD & CKD-EPI	3	21.3(-14.9-57.6)			8.5(-25.0-41.9)		

### Distribution by study design, sampling technique and sample size

Result showed a statistically significant weak and negative correlation between CKD prevalence rate and sample size (r = -.213, p = 0.019). Studies with smaller sample sizes reported higher rates. There was a statistically significant difference in CKD prevalence rate between cross-sectional studies and cohorts, with cross-sectional studies reporting a higher prevalence rate (F= 6.239, p = 0.014). There was a statistically significant difference in the reported CKD prevalence rate between studies that employed randomization and those that did not (F = 8.810, p = .004). Studies which employed non-random sampling reported statistically and epidemiologically significant prevalence rates compared to studies which employed random sampling (Table 3).

### Distribution by region

Distribution of the burden of CKD in the region differed epidemiologically but not statistically (F = 2.096, P =0.128); East Africa's rate (26.95%) was higher than those of West Africa (20.39%) and Southern Africa's (19.3%). Epidemiologically, the difference in prevalence by 7% is considered significant^15^ (Table 3).

### Distribution of CKD by the study population

There was a statistically significant difference in CKD prevalence by population (F=2.547, p=.019), with the burden being highest in sickle cell patients (38.07%), followed by diabetic patients (32.60%), people living with HIV (27.62%), and hypertensive patients (21.12%).

### Distribution by the method of estimating GFR

There was no statistically significant difference in CKD prevalence by the method of estimating GRF (F=1.089, p =.366). However epidemiological significance difference was found between Schwartz approach (35.03%), and each of Cockcroft-gault (24.61%), CKD-EPI (22.68%), and MDRD (20.96%).

### Trend in the distribution of CKD in Africa

**Figure 4:**
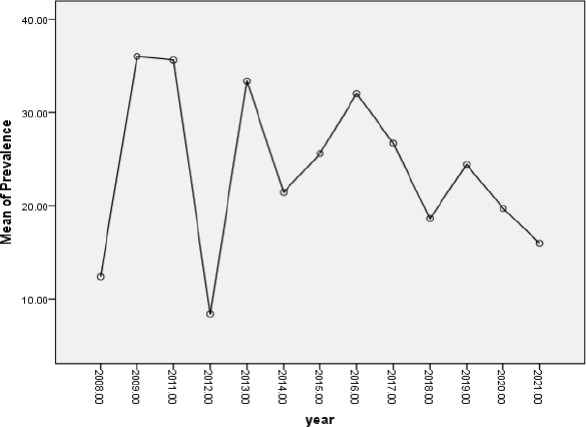
Trend of the prevalence distribution of CKD in Africa

The highest prevalence of CKD was recorded 2009, 2011 and 2013, with prevalence of 36, 35.6%, and 33.4%, respectively. The lowest were in 2012 and 2008, with values of 8.4% and 12.4%, respectively. Since 2019, there was a progressive decline with the current prevalence being 16% (Figure 4). Study characteristics

## Discussion

In this study, the pooled prevalence of CKD in sub-Saharan Africa was 18% with a burden of over 174 million. This implies that it is at an alarming rate and people should be enlightened on the need to start management at an early stage through routine medical checkups. Our value agrees with the systematic review done by Ajayi et al^19^ with a prevalence of 17.8%. Also, George and colleagues^20^ review obtained a CKD prevalence of 14.3% in the general population and 36.1% in the high-risk population. Contrary to our study, El-hafeez and co1 in their global review obtained a lower CKD prevalence of 13.4, with a sub-Saharan value pegged at 14%. Hill et al.^21^ in their global systematic review and meta-analysis, reported a lower pooled prevalence of 13.4%. According to them, global stratification of that result revealed that developed areas like Europe, the USA, Canada and Australia have higher rates of CKD prevalence compared to growing economies like in sub-Saharan Africa.

Unlike in sub-Saharan Africa with a heterogeneous CKD distribution, prevalence in Europe tends to be lower and homogeneous being 8.9% in the Netherlands, 6.8% in Italy, 5.2% in Portugal, 4.7% in Spain, 3.3% in Norway^1^. CKD contributes large health and economic burden within sub-Saharan Africa, in consonance with high poverty levels and out-of-pocket expenditure for healthcare in the region^22^. The economic burden of CKD could be associated with the late presentation of health services conditions and prevalence data impact across the region^23^,^22^. Several studies' findings suggested that a limited fraction of the sub-Saharan population can afford hemodialysis^24^,^25^ and by the year 2030, a large percentage of patients with CKD will be from developing nations^26^.

Although the result of this meta-analysis reported that age could affect CKD distribution in sub-Saharan Africa, there was no indication from the study that gender has any significant effect on this distribution, however in the setting of SSA where majority of the population is under 30 years, it is likely that there is not enough variety in ages to show significance. Additionally, this highlights the grave impact such a high burden of CKD will have on economic development. Contrarily, the risk of CKD development, progression, and complications is well-known to be influenced by gender and age^1^. Males are more likely to high level of CKD severity, although females tend to have a higher prevalence of the condition. The reason for the inconsistency could be due to the paucity of data on its prevalence by age and gender^1^. The finding that older adults recorded higher CKD prevalence compared to young adults collaborates with previous studies^27^,^28^,^29^. Such disparities in the distribution of CKD prevalence across gender and age are reflected also in its management where there have been gender disparities in access to CKD treatment^29^. To bridge the gender gap of CKD prevalence within the region, it is essential to promote equal access to renal function-related healthcare^29^.

The study highlights that, for an unbiased estimate of CKD prevalence in sub-Saharan Africa, the methods employed in determining CKD prevalence and other factors such as sample size, and study population should be taken into consideration and adjusted for when estimating CKD burden in the region1. Similar studies^30^,^19^ showed that, with the vast majority of studies done in urban settings, the methodology and population structure adopted to conclusively define CKD prevalence in sub-Saharan Africa might not be representative of the entire population evidenced by low study quality and detection bias. The lack of reliable measures of kidney function among cohort studies studying the prevalence of kidney disease in sub-Saharan Africa poses a great threat to the validity and reliability of the data presented^30^. Nonetheless, for the prevalence estimation objective, the cross-sectional design is a better option for optimizing CKD prevalence in the region as it remains the most relevant design when assessing the prevalence of the disease^31^. However, when data from cohort studies are included in the summary estimate, a correction factor should be developed to account for the likelihood of underestimation of CKD burden in these studies.

The epidemiological distribution of CKD in sub-Saharan Africa from the study revealed that East Africa has a higher health burden compared to other areas within the region. This could imply that resource allocation from international bodies towards relieving the CKD burden in the region will favour the region towards achieving health equity. Population groups in various regions of the world are adversely affected by CKD in different ways, most likely due to variations in comorbidities, underlying demographics, and availability of healthcare services. Prevalence and debilitating effects of CKD should spur significant efforts to create and put into place efficient prevention and treatment measures intended to ensure health equity on a global level^32^,^33^.

The burden of CKD in sub-Saharan Africa as revealed by this study could be associated with different conditions including diabetes, HIV, and hypertension. Several studies^31^,^33^,^1^,^34^ have also reported high CKD prevalence among people with high body-mass index, high sodium diet, diabetes, hypertension and HIV. CKD is a notable persistent HIV consequence recorded in sub-Saharan Africa, making HIV treatment in sub-Saharan Africa a significant concern for global health^29^. In sub-Saharan Africa, diabetes and hypertension dominate the spectrum of CKD causes. These comorbidities are influenced by the study population's variability, racial diversity, cultural backgrounds and nutritional condition^1^,^6^,^36^.

Our study showed that Schwartz was used (at 35.03%), Cockcroft-gault followed at 24.61%, CKD-EPI at 22.68%, and MDRD at 20.96%. This implies that for persons with the same demographics and race, when different GFR estimation methods are used, you would get varying values. This is consistent with Shiferaw et al^37^ which observed variation of CKD prevalence per diagnostic criterion. In a similar study, epidemiological differences were reported in approaches employed in estimating GFR among CKD patients in sub-Saharan Africa with the Schwartz method used mostly^38^. Globally, different equations have been used to estimate GFR among CKD patients^26^, with MDRD and CKD-EPI being the mostly used equations^26^,^39^. GFR estimation has been adjusted for ethnicity and gender difference severally which provides variable-specific differences between CKD prevalence^21^,^39^. When ethnic variables are included with these different equations, it provides a prevalence report that is adjusted for ethnicity-specific differences in different regions of the world^39^. CKD is a public health concern with an increasing global burden^32^,^40^ and a leading non-communicable determinant of mortality^33^. Approximately 800 million people worldwide have kidney disease^33^. In Low- and middle-income countries, CKD represents a large burden with limited resources available to mitigate its risks and effects^41^. This makes CKD a disease of public health concern which forms a relevant background to the United Nations' sustainable development goal among other NCDs to reduce premature mortality from non-communicable diseases globally by one-third in 2030^42^. Efforts to reduce inequalities among CKD patients and promote universal health coverage would prioritize providing equitable access to affordable care^41^.

There seems to be progressive decline in the prevalence of CKD since 2019. The reason for this decline is not clear. However there are several efforts employed to contain the CKD epidemic in Africa^43^. It is possible that such efforts are beginning to yield fruits. In that case more efforts need to be intensified to expand their benefits in order to continue this downward trend that can help to reduce the burden of CKD in Africa bearing in mind that Africans are prone to kidney disease due to high prevalence of apolipoprotein a major risk factor for kidney disease, observed in populations of sub-Saharan African extraction^44^.

The strength of this study lies in the use of over 90 studies and in the application of meta-analysis to synthesize the prevalence and distribution of CKD in sub-Saharan Africa. The considerable degree of heterogeneity (I2 = 98.74%) and the fact that we did not measure the level of agreement between the two reviewers who undertook title and abstract screening constitute some limitations to the study. According to the Cochrane Handbook for Systematic Reviews of Interventions, I2 values of 0-40% may indicate low heterogeneity, 30-60 % may indicate moderate heterogeneity, 50-90% may indicate substantial heterogeneity and 75-100% may indicate considerable heterogeneity^14^. However, we ensured that disagreement was resolved by the experienced reviewer.

## Conclusion

There appears to be progressive decline in the prevalence of CKD in Africa since 2019. However, CKD remains a public health issue in sub-Saharan Africa and the distribution varies widely according to region, study population and method of estimating glomerular filtration rate.
